# Impact of therapeutic strategy on disease‐free and overall survival of early‐stage cervical cancer: Surgery alone versus preoperative radiation

**DOI:** 10.1002/cnr2.1823

**Published:** 2023-04-19

**Authors:** Katia Mahiou, Laura Vincent, Karine Peignaux‐Casasnovas, Hélène Costaz, Marie‐Martine Padeano, Alix Amet, Sandrine Dabakuyo, Ariane Mamguem Kamga, Leila Bengrine‐Lefevre, Charles Coutant

**Affiliations:** ^1^ Department of Surgical Oncology Georges François Leclerc Cancer Center –UNICANCER Dijon France; ^2^ Department of Radiotherapy Georges François Leclerc Cancer Center – UNICANCER Dijon France; ^3^ Department of Education University of Burgundy–Franche Comté Dijon France; ^4^ Côte d'Or Breast and Gynaecological Cancer Registry Georges François Leclerc Cancer Center –UNICANCER Dijon France; ^5^ Department of Medical Oncology Georges François Leclerc Cancer Center –UNICANCER Dijon France

**Keywords:** early cervical cancer, radiotherapy, surgery

## Abstract

**Background and Objectives:**

There is no international consensus for management of early‐stage cervical cancer (ESCC). This study aimed to retrospectively investigate disease‐free survival (DFS) and overall survival (OS) in patients with ESCC according to the therapeutic strategy used, surgery alone versus preoperative radiation following by surgery.

**Methods:**

Data were retrospectively collected from 1998 to 2015 using the Gynecological Cancer Registry of the Côte d'Or. The inclusion criteria were FIGO 2018 ≤ IB2; squamous cell carcinoma, adenocarcinoma or adenosquamous type. Survival curves were compared using the log‐rank test.

**Results:**

One hundred twenty‐six patients were included. Median survival was 90 months. There was no significant difference in DFS (HR = 0.91, 95%CI [0.32–2.53], *p* = 0.858) or in OS between surgery alone versus preoperative radiation following by surgery (HR = 0.97, 95%CI [0.31–2.99], *p* = 0.961). In the subgroup of patients with stage ≥IB1, there was no significant difference in DFS (HR = 3.26, *p* = 0.2) or in OS (HR = 3.87, *p* = 0.2).

**Conclusion:**

Our study did not identify any difference in survival according to the treatment strategy. Preoperative radiation following by surgery can be an alternative to surgery alone for ESCC.

## 1 INTRODUCTION

Cervical cancer accounts for 2920 new cancer cases per year, and caused 1117 deaths in 2018 in France.^1^ According to the 2018 International Federation of Gynaecology and Obstetrics (FIGO) classification, early‐stage cervical cancers are those that are at or below stage IB2.^2^

Currently, the management of early‐stage cervical cancer is based on the European guidelines updated in 2018, as well as on the FIGO guidelines.^2^, ^3^ For cancers at 2018 FIGO stage IA1, conization alone can be performed in women desiring fertility preservation.^4^ In case of positive margins, a repeat conization should be performed. In case of positive margins or LVSI on the surgical specimen, radical hysterectomy should be performed.

Lymph node staging is not indicated in T1a1 LVSI‐negative patients but can be considered in T1a1 LVSI‐positive patients, by sentinel lymph node (SLN) biopsy. The SENTICOL‐3 study investigated 3‐year disease‐free survival (DFS) and health‐related quality of life (HRQoL) after SLN mapping or SLN plus pelvic lymph node dissection (LND) in patients with early‐stage cervical cancer.^5^ For stage IA2, in the absence of LVSI on the conization specimen, hysterectomy of type A according to the Querleu‐Morrow classification or type I of the Piver and Rutledge classification may be performed.^6^, ^7^ Conversely, in case of LVSI, Querleu‐Morrow type B or Piver and Rutledge type II hysterectomy should be performed.^6^, ^7^ In women desiring fertility preservation, two therapeutic options are possible: (i) conization with LND; or (ii) trachelectomy using an abdominal, vaginal or minimally‐invasive approach, associated with LND. Parametrial resection is not recommended. As for stage IA1, SLN alone cannot be recommended outside prospective clinical trials.^5^ Routine completion hysterectomy is not recommended after conservative management if the lymph nodes are negative.

The management of FIGO stage IB1 cancers depends on the risk category of the patient. Tumor size (greater or less than 2 cm), maximal stromal invasion and LVSI are used to classify patients as high, intermediate, or low‐risk. According to the European Society of Gynaecological Oncology (ESGO), European Society for Radiotherapy and Oncology (ESTRO) and European Society of Pathology (ESP), Querleu type B1 hysterectomy should be performed in low‐risk patients, Querleu type B2 in intermediate‐risk, and type C1 in high‐risk patients. Ovarian preservation should be offered to premenopausal patients with squamous cell carcinoma and human papillomavirus (HPV)‐related adenocarcinoma. SLN biopsy with LND should be performed in these patients. Trachelectomy is an option in patients desiring fertility preservation. Radiotherapy associated with brachytherapy alone can be considered in case of unfavorable prognosis or anticipated high surgical morbidity. For intermediate and high‐risk patients, preoperative brachytherapy followed by type A surgery is an acceptable option. For FIGO stage IB2, the FIGO recommendations propose primary surgery or primary radiation therapy, depending on the patient's comorbidities and local availability and expertise in the different treatment options.

Surgery plays a central role in the management of early‐stage cervical cancer. Retrospective data regarding preoperative brachytherapy in early‐stage cervical cancer suggest that it is effective and well tolerated.^8^, ^9^, ^10^ In recent years, therapeutic strategies for early‐stage cervical cancer have evolved. For tumors measuring less than 2 cm, fertility preservation and de‐escalation of therapy are now major issues at stake in the therapeutic management of young patients. At present, there is no international consensus regarding the *gold standard* for management of early‐stage cervical cancer.

In this context, using data from the Breast & Gynecological Cancer Registry of the Côte d'Or, collected between 1998 and 2015, this study aimed to retrospectively investigate overall survival (OS) and DFS in patients with cervical cancer of stage IA1, IA2, IB1 or IB2 (FIGO 2018) according to the therapeutic strategy used, namely, surgery alone versus preoperative radiation therapy.

## 2 MATERIALS AND METHODS

### 2.1 Data collection

Multicenter data on patients with early‐stage cervical cancer were retrospectively collected from January 1998 to December 2015 using the Breast Cancer Registry of the Côte d'Or. The management of patients with breast and gynecological cancer in the department is multidisciplinary and is centralized mainly in Dijon, the largest city of the department, which has a comprehensive cancer care center (60% of patients of the registry), a University Hospital and some private hospitals. This registry is the only French registry dealing specifically with breast and gynecological cancers. Since 1982, the registry has been collecting data on all cases of breast and gynecological cancers occurring in residents of the Côte d'Or Departement in Eastern France.

Because of the inclusion period, we chose to reclassify patients according to the 2018 FIGO classification.

To be eligible, patients had to meet the following inclusion criteria: be ≥18 years old; have a first diagnosis of histologically proven squamous cell carcinoma, adenocarcinoma, or adenosquamous carcinoma of stage IB2 or lower according to FIGO 2018; had data regarding the treatment received, including primary surgery or preoperative radiation therapy. Patients meeting any one or more of the following criteria were excluded: history of pelvic radiation, concomitant radiochemotherapy, pathological nodes in histology, adjuvant radiotherapy, total radiation dose <50 Gy, >66 Gy or not documented.

The surgery group comprised patients who underwent surgery (hysterectomy, trachelectomy or conization) as the only form of treatment for early‐stage cervical cancer. The radiation therapy group comprised patients who had radiation therapy (brachytherapy and/or radiotherapy) followed by surgery (hysterectomy, trachelectomy or conization).

### 2.2 Statistical analysis

Categorical variables are presented as number and percentage, and quantitative variables as mean and standard deviation or median and interquartile range. Variables were compared using the Chi^2^ or Fisher's exact test for qualitative variables and the student's t‐test for quantitative variables.

The Kaplan–Meier method was used to describe disease‐free survival (DFS) and overall survival (OS). Survival was determined for the entire population and subgroup analysis was performed for ≥ IB1 population and in patients who had hysterectomy. Survival curves were compared using the log rank test. Adjusted survival curves were estimated using the direct adjusted survival method via a Cox model adjusted for FIGO stage and pelvic lymphadenectomy. Test results were considered significant when the *p*‐value was <.05. Recurrence was defined as any lesion identified on biopsy and histologically diagnosed as recurrent invasive cervical cancer. DFS was defined as the time (in months) from the date of the initial histological diagnosis to the date of the first histological diagnosis of recurrence or death. Patients who did not have recurrence or death were censored at the date of last follow‐up. OS was defined as the time (in months) from the date of the initial histological diagnosis to the date of death (all causes). Patients who were still alive at the cut‐off date were censored.

Survival data were available for all patients in 2018, allowing for at least 3 years of follow‐up for all patients. Survival curves were compared using the log rank test. All statistical analyses were performed using RStudio Team (2020) software. RStudio: Integrated Development for R (RStudio, PBC, Boston, MA) http://www.rstudio.com/ and SAS version 9.4 (SAS institute Inc., Cary, NC, USA).

## 3 RESULTS

### 3.1 Patients features

**FIGURE 1:**
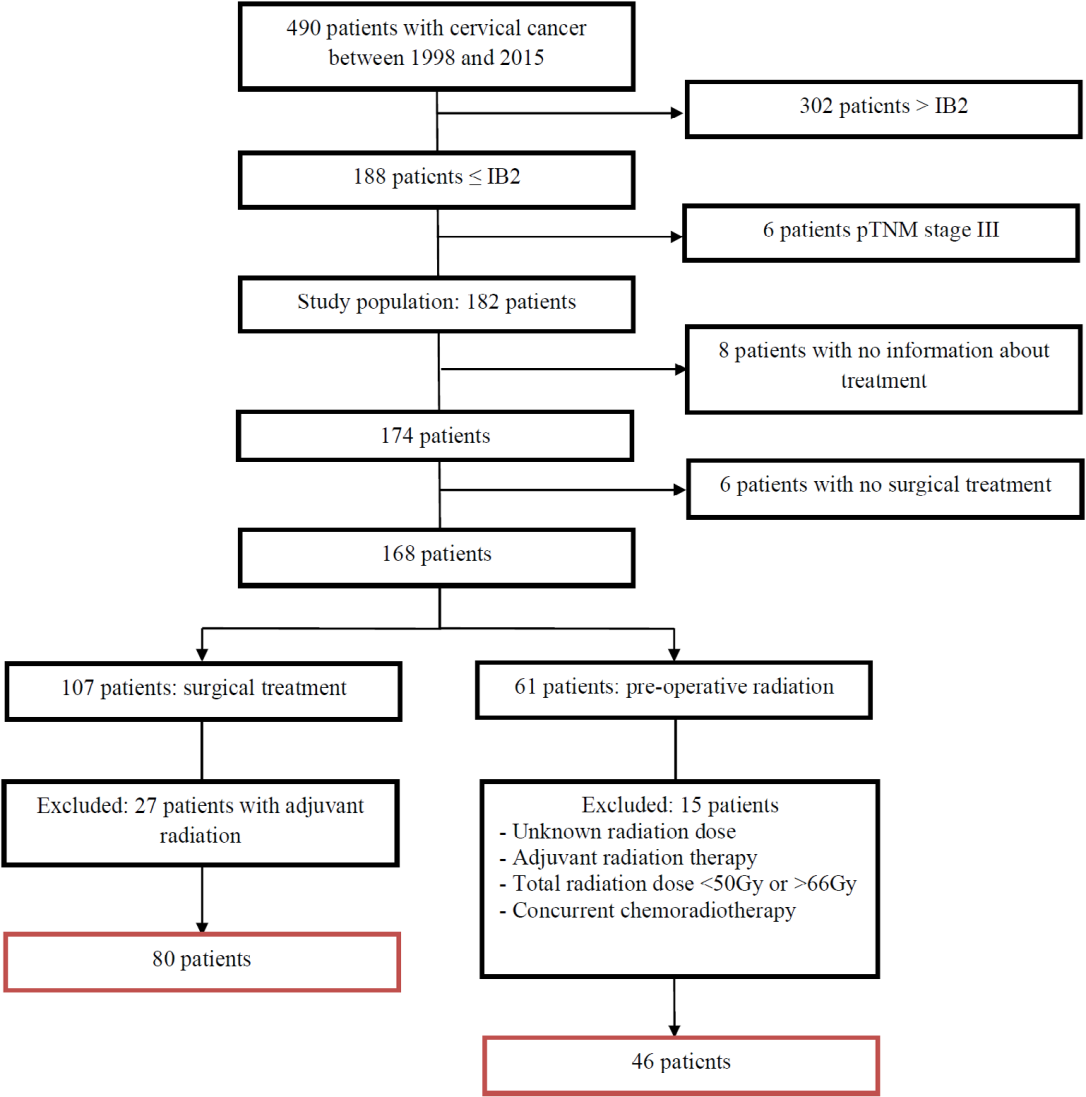
Flow‐chart.

Among 490 patients with early‐stage cervical cancer, 302 were excluded because the FIGO stage was greater than IB2, 6 were excluded because they had pTNM stage III, 8 were excluded due to missing data about the treatments received, 6 because they did not undergo surgery, 27 because they received adjuvant pelvic radiation after primary surgery, 3 because they received concomitant radiochemotherapy, and 12 because the radiation dose received was either <50 Gy, >66 Gy or not documented (Figure 1).

Finally, 126 patients were included in the present analysis: 80 patients in the surgery group, and 46 in the radiation group. Average age at diagnosis was 46.9 years; 65 patients had stage IA tumors, and 61 had stage ≥IB1. The predominant histological type was squamous cell carcinoma (84.1%). Prior conization was performed in 60.3%. Hysterectomy was performed in 84.1% of patients (36.8% by laparotomy, 28.3% minimally invasive surgery (coelioscopy or robot‐assisted) and 19.8% by the vaginal approach). Half the patients (50.8%) underwent pelvic LND, while 4.8% had SLN plus LND. Finally, 11 recurrences were observed, most of which were local pelvic recurrence (72%) (Table 1).

**TABLE 1:** Characteristics of the study population (*N* = 126).

	Total	Surgery	Radiation	*p‐*value
Number	126	80 (63.5%)	46 (36.5%)	
Mean age, years (±sd)	46.9 ± 13.9	44.5 ± 12.5	51 ± 15.5	.02
Median follow‐up (1st–3rd)	90 (47–148)	72 (45.7–121.2)	135.5 (72.2–187.5)	.7
FIGO (2018)				<.05
IA	65 (51.6%)	58 (72.5%)	7 (15.2%)	
≥IB1	61 (48.4%)	22 (27.5%)	39 (84.8%)	
Tumor size				<.05
<2 cm	80 (63.5%)	64 (80%)	16 (34.8%)	
≥2 cm	21 (16.7%)	4 (5%)	17 (36.9%)	
Unknown	25 (19.8%)	12 (15%)	13 (28.3%)	
Gestation				.65
G0	10 (7.9%)	7 (8.8%)	3 (6.5%)	
G1–G3	64 (50.8%)	35 (43.7%)	29 (63%)	
≥G4	20 (15.9%)	12 (15%)	8 (17.4%)	
Unknown	32 (25.4%)	26 (32.5%)	6 (13.1%)	
Tumor type				.57
Squamous cell	106 (84.1%)	69 (86.3%)	37 (80.4%)	
Adenocarcinoma	18 (14.3%)	10 (12.5%)	8 (17.4%)	
Adenosquamous	2 (1.6%)	1 (1.2%)	1 (2.2%)	
LVSI				.57
Yes	14 (11.1%)	8 (10%)	6 (13%)	
No	110 (87.3%)	71 (88.7%)	39 (84.8%)	
Unknown	2 (1.6%)	1 (1.3%)	1 (2.2%)	
Previous conization				.07
Yes	76 (60.3%)	49 (61.3%)	27 (58.7%)	
No	50 (39.7%)	31 (38.7%)	19 (41.3%)	
Surgery				
Hysterectomy	106 (84.1%)	61 (76.3%)	45 (97.8%)	<.05
Open surgery	39 (36.8%)	15 (24.6%)	24 (53.3%)	<.05
MIS	30 (28.3%)	23 (37.7%)	7 (15.6%)	.07
Vaginal	21 (19.8%)	18 (29.5%)	3 (6.7%)	.01
Unknown	16 (15.1%)	5 (8.2%)	11 (24.4%)	
Trachelectomy	5 (4%)	4 (5%)	1 (2.2%)	.65
Conization	15 (11.9%)	15 (18.7%)	0 (0%)	<.05
Lymphadenectomy				
Pelvic LND	64 (50.8%)	27 (33.7%)	37 (80.4%)	<.05
Pelvic LND + SLN	6 (4.8%)	6 (7.5%)	0 (0%)	.08
None	56 (44.4%)	47 (58.8%)	9 (19.6%)	
Number of pelvic lymph nodes per patient				
Mean	9.8	12.8	7.6	<.05
Type of parametrectomy (Hysterectomy and trachelectomy, *n* = 111)				0.08
Piver I, Querleu A	48 (43.2%)	33 (50.8%)	15 (32.6%)	
PiverII, III, Querleu B‐C	60 (54.1%)	31 (47.7%)	29 (63%)	
Unknow	3 (2.7%)	1 (1.5%)	2 (4,4%)	
P16 protein				<.05
Positive	45 (35.7%)	37 (46.3%)	8 (17.4%)	
Unknown	81 (64.3%)	43 (53.7%)	38 (82.6%)	
First recurrence (*n* = 11)				.66
Pelvic recurrence	8 (72.7%)	4 (100%)	4 (57.1%)	
Metastasis	2 (18.2%)	0 (0%)	2 (28.6%)	
Pelvic recurrence and metastasis	1 (9.1%)	0 (0%)	1 (14.3%)	
Deaths (*n* = 25)				
	25 (19.8%)	11 (13.8%)	14 (30.4%)	

Abbreviations: LND, lymph node dissection; LVSI, lymphovascular space invasion; MIS, mini‐invasive surgery; Sd, standard deviation, SLN, sentinel lymph node.

The majority (76%) of patients in the radiation therapy group receive neoadjuvant brachytherapy alone, 21.8% received brachytherapy plus radiotherapy, while only one patient received radiotherapy alone. The mean total dose of radiation received (by brachytherapy or radiotherapy) was 59.1 Gy.

Complete histological response was observed on the surgical specimen in 60.9% of patients. The average time from the end of radiation therapy to surgery was 5.8 weeks (Table 2).

**TABLE 2:** Radiation therapy characteristics.

	*n* = 46 (%)
Radiation type	
Brachytherapy only	35 (76)
External radiotherapy	1 (2.2)
Brachytherapy and radiotherapy	10 (21.8)
Total dose of external radiotherapy	
Mean (Gy)	45.1
≤ 40Gy	2 (18.2)
>40Gy	9 (81.8)
Total dose of brachytherapy	
Mean (Gy)	49.4
<60Gy	19 (42.2)
≥60Gy	26 (57.8)
Total dose of radiation	
Mean (Gy)	59.1
<55Gy	5 (10.9)
≥55Gy–<60Gy	5 (10.9)
≥60Gy	36 (78.2)
Number of brachytherapy sessions	
1	31 (68.9)
2	14 (31.1)
Type of brachytherapy	
Low‐dose rate	35 (77.8)
Pulse dose rate	10 (22.2)
Pathological complete response	
Yes	28 (60.9)
No	17 (36.9)
Unknown	1 (2.2)
Time between radiation and surgery	
Mean (weeks)	5.8

Abbreviation: Gy, Gray.

### 3.2 Survival analysis

Median survival in the whole population was 90 months (47–148); 72 months for those receiving primary surgery, and 135.5 months for those receiving primary radiation therapy. For the subgroup of ≥IB1 patients, it was 87 months (46–167).

**FIGURE 2:**
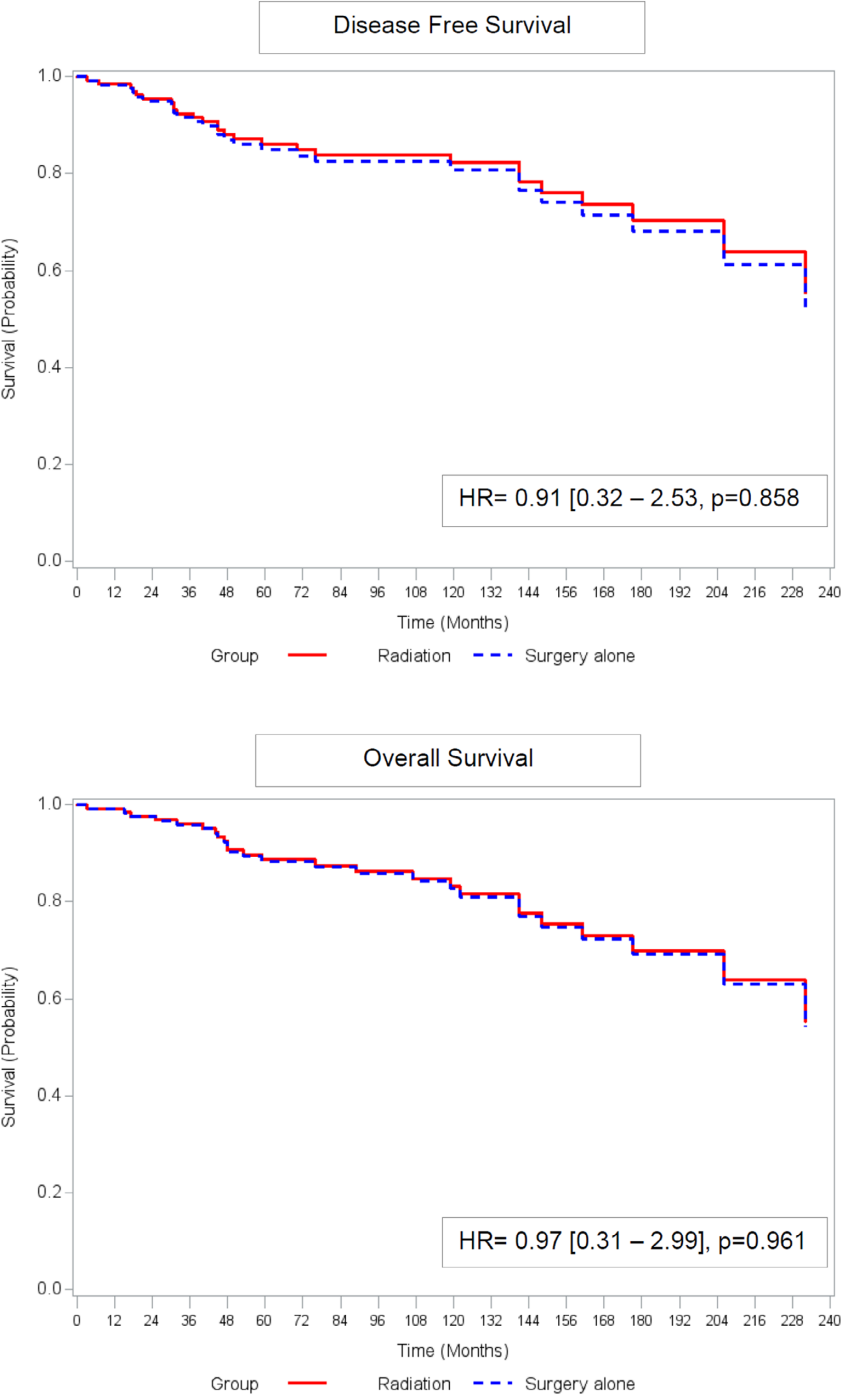
DFS and OS in the overall population adjusted for FIGO stage and pelvic lymphadenectomy (Surgery vs. preoperative radiation).

**FIGURE 3:**
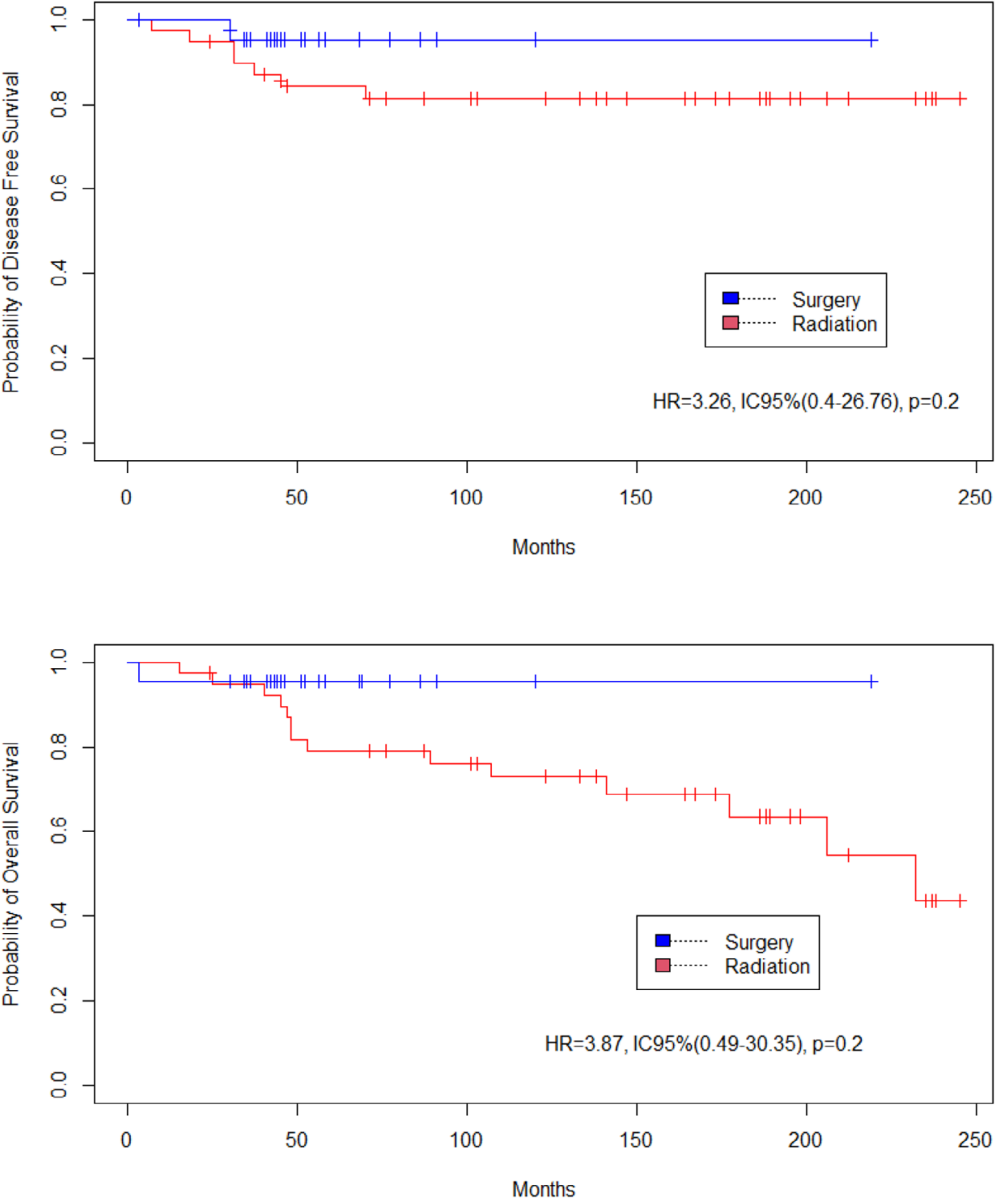
DFS and OS for stage ≥ IB1 (Surgery vs. preoperative radiation).

**FIGURE 4:**
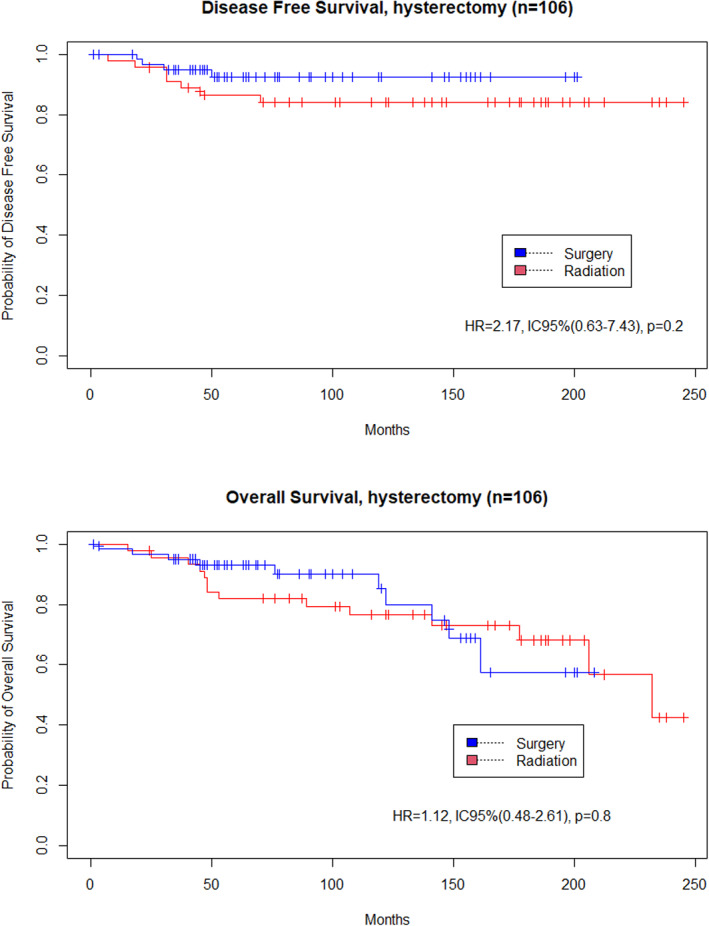
DFS and OS for patients with hysterectomy (*n* = 106) (Surgery vs. preoperative radiation.

There was no significant difference in DFS between the surgery and radiation groups (HR = 0.91, 95%CI [0.32–2.53], *p* = .858). Similarly, there was no significant difference in OS between groups (HR = 0.97, 95%CI [0.31–2.99], *p* = .961) (Figure 2). In the sub‐group of patients with stage ≥IB1, there was no significant difference in DFS (HR = 3.26, 95%CI [0.4–26.76], *p* = .2) or in OS (HR = 3.87, 95%CI [0.49–30.35], *p* = .2) between groups (Figure 3). In the sub‐group of patients who underwent hysterectomy, there was no significant difference in DFS (HR = 2.17, 95%CI [0.63–7.43], *p* = .2) or in OS (HR = 1.12, 95%CI [0.48–2.61], *p* = .8) (Figure 4).

## 4 DISCUSSION

This study did not find any significant difference in DFS or in OS after surgery alone versus preoperative radiation therapy for patients who presented with early‐stage cervical cancer. These results are in line with the findings of the only prospective randomized study to date, namely the study by Landoni et al. comparing radical surgery versus radiation for early‐stage cervical cancer.^11^, ^12^ In their study, 343 patients with stage IB and IIA cervical carcinoma were randomized to receive radical surgery or radical radiotherapy (radiation only). Adjuvant radiotherapy was administered in 54% of patients with a tumor diameter less than or equal to 4 cm and in 84% of patients with a tumor diameter greater than 4 cm. In 2017, the results were updated with almost 20 years of follow‐up data, and OS was 72% and 77% (*p* = .28) for surgery and radiotherapy, respectively.^12^ The update study also investigated the impact of histological type on OS and found that when selecting patients with adenocarcinoma histology, 20‐year OS was better in patients who underwent surgery compared to those randomized to radiotherapy (71% vs. 47%, respectively, *p* = .09). In our study, the low number of patients with adenocarcinoma histology precluded showing any difference in terms of survival for this histological type. Currently, the histological type does not influence the chosen therapeutic strategy. We therefore chose to keep adenocarcinomas in the final analysis.

The choice between surgery alone or preoperative radiation was made during multidisciplinary consultation meetings according to comorbidities and histological results at diagnosis. This non‐standardized choice may constitute a bias in our study.

Currently, adjuvant radiotherapy is recommended for intermediate risk lesions according to Sedlis criteria. During the period of our study, adjuvant radiotherapy was discussed for high risk or in case of more advanced stage on the surgical specimen. The criteria used to determine whether pelvic radiation therapy with chemotherapy should be performed after radical hysterectomy is as follows: presence of LVSI, depth of invasion, and tumor size.^13^

To limit bias between groups, patients who received adjuvant radiotherapy were excluded from the analysis. Regarding preoperative radiation, to limit the bias related to the radiation dose received, we excluded patients who received less than 50Gy because they were potentially undertreated, and we also excluded patients who received more than 66Gy because they did not have adjuvant surgery because of the radiation dose and the risks of significant morbidity.

Although both therapeutic strategies seemed to be equivalent in terms of survival, there were differences in terms of morbidity and short‐ and long‐term complications. In a study by Lamblin et al.^14^ among 45 patients with stage IB1 (FIGO 2009) cervical cancer, early and late complications, especially urinary complications, seemed to be greater in patients receiving a combination of brachytherapy plus surgery, compared to surgery alone, albeit without reaching statistical significance (*p* = .22). Nowadays, technological advances in techniques for pelvic radiation have made it possible to limit these complications. In France, since 2005, brachytherapy optimized by 3D imaging, such as pulse‐dose rate, has almost halved grade 3 and 4 complications at 2 years compared to 2D brachytherapy (14.6% versus 8.9% of grade 3 and 4 complications in the group with FIGO IB1 lesions).^8^ The EMBRACE study is currently prospectively evaluating local control, early and late morbidity and quality of life of patients with cervical cancer treated by image‐guided adaptive brachytherapy .^15^, ^16^

When surgery is the chosen treatment option, radical colpohysterectomy with pelvic LND is the reference in early‐stage cervical cancer. In our study, most patients underwent radical hysterectomy (54.1%) and pelvic lymphadenectomy was recommended for stages ≥IA2. Due to comorbidities and operative difficulties, some patients did not benefit from pelvic lymphadenectomy (2 patients in the radiation group). Because of the inclusion period, the SLNs performed were in study protocol (SENTICOL).

The main complication of this surgery is lesions of the urinary and digestive tracts, with bladder dysfunction, and impaired sexual and digestive function related to lesions of the autonomic nervous system.^17^ With a view to de‐escalating therapy and preserving fertility, several surgical techniques appear comparable, in terms of DFS and OS, with considerable reductions in short‐ and long‐term complications.^18^ Radical trachelectomy is the therapeutic option retained in patients desiring fertility preservation. A systematic review of the literature by Nezhat et al.^18^ that included 65 studies and evaluated reproductive and oncologic outcomes after fertility‐sparing surgery, reported a mean cancer recurrence rate of 3.2% with no significant different across surgical approaches (*p* = 0.659) (laparoscopic, mini‐invasive, or vaginal approaches).

Parametrial involvement is a major prognostic factor in early‐stage cervical cancer. Using retrospective data from the SENTICOL I and II studies from 2005 to 2012, Benoit et al. evaluated parametrial invasion in patients with early‐stage cervical cancer (FIGO 2018 classification stage IA with LVSI to IIA1). Among 211 patients included, 11 (5.2%) presented histologic parametrial involvement (7 [63.6%] were FIGO 2018 stage IB1 and 4 stage IB2 [36.4%]).^19^ Parametrial involvement is a source of short and long‐term complications. Piver type III radical hysterectomy is associated with significantly more complications (especially urologic morbidity) compared to Piver type II (28% vs. 13%), but did not affect 5‐year OS (81% vs. 77%) or DFS (75% and 73%).^6^, ^20^ Several risk models have been studied to identify subgroups of patients who might be eligible for less radical therapy.^19^, ^21^ Currently, the ongoing randomized, phase III SHAPE study is prospectively comparing the efficacy and safety of radical hysterectomy with pelvic LND versus simple hysterectomy and pelvic node dissection in patients with low‐risk early‐stage cervical cancer.^22^

In this context, conization with SLN biopsy appears to be an attractive therapeutic option in patients with IB1 lesions desiring fertility preservation.^23^ In our study, 15 patients had conization only as the surgical treatment. All 15 had stage IA lesions, and none had LVSI. One of the 15 patients had concomitant LND by laparoscopy. None of the 15 presented recurrence during follow‐up. In the literature, conization alone or together with LND seems to be safe in terms of survival, if there is a minimum of 2 mm clear margins, and the conization specimen measures at least 20 mm.^4^, ^24^, ^25^

Prior to 2018, when hysterectomy was indicated, all surgical approaches could be envisaged, and assumed to yield equivalent oncological outcomes, but recommendations were based on retrospective literature data. The LACC study was the first international, multicenter, prospective, randomized trial to aim to demonstrate the non‐inferiority of minimally invasive surgery (laparoscopic or robot‐assisted radical hysterectomy) compared to open abdominal radical hysterectomy.^26^ Between June 2008 and July 2017, the trial recruited patients who had FIGO 2009 stage IA1 with or without LVSI, stage IA2 or stage IB1; and who underwent type II or III radical hysterectomy (according to the Piver classification^6^). Minimally invasive surgery was associated with a lower rate of DFS compared to open surgery at 3 years (91.2% vs. 97.1%, HR 3.74, 95%CI 1.63–8.58), and minimally invasive surgery was also associated with lower OS at 3 years (93.8% vs. 99.0%; HR = 6.00; 95%CI [1.77–20.30]).

In 2019, the American clinical practice guidelines released by the NCCN for the management of cervical cancer took into account the findings of this study by Ramirez et al., but did not issue any recommendation about which surgical approach should be preferred.^27^ Nevertheless, expert consensus recommended open radical hysterectomy for lesions ≥2 cm. In our study, the small sample size did not enable us to show any survival difference according to the surgical approach chosen for hysterectomy (with 2 recurrences in those with minimally invasive surgery, and 2 in the laparotomy group, *p* = .8; and also two deaths in each group, *p* = .2).

The morbidity associated with pelvic LND is not negligible, especially when associated with pelvic radiation. Lymph node status is a prognostic factor, and a major contributing factor to the decision about adjuvant therapy. In the 2018 FIGO classification, pelvic lymph node metastases (micro‐ and macro‐metastases) qualified the disease as stage IIIC1, independently of the tumor size.^2^ SLN biopsy is safe and has been shown to yield a low rate of false‐negatives (3.6%).^28^ It also significantly reduces the complications of surgery, notably lymphoedema of the lower limbs (87% vs. 42%, *p* = .03).^29^ In a study of data from the SENTICOL study, 60.5% of detected SLNs were in the external iliac and interiliac basin, while a substantial proportion were in unusual locations, namely 19.6% in the common iliac, 11.8% in the para‐aortic, and 6% in the parametrium.^30^ Ultrastaging of SLNs makes it possible to identify micrometastases and isolated tumor cells, although the impact of these findings remains to be demonstrated.^31^, ^32^

Surgery and radiotherapy are the mainstay of treatment for early‐stage cervical cancer. However, in years to come, new treatment options will be developed. Neoadjuvant chemotherapy makes it possible to reduce the tumor size, and in this context, may be a viable treatment option for women desiring fertility preservation, by enabling conservative surgery.^33^ Response to neoadjuvant chemotherapy has been shown to be an independent prognostic factor for survival.^34^ The CONTESSA/NEOCON‐F study is an ongoing prospective, multicenter, phase II trial evaluating the safety of neoadjuvant chemotherapy followed by fertility‐sparing surgery in young women with stage IB2 lesions.^33^ This ongoing study should help to achieve standardization of management for young patients desiring fertility preservation. While chemotherapy appears to enable de‐escalation of surgical therapy, vaccination is also among the new options currently being investigated for the treatment of cervical cancer, notably in the VOLATIL study.^35^ This ongoing phase II study will evaluate the clinical impact and immunological efficacy of combining a CD4‐help T‐inducer vaccine (universal cancer peptide vaccine, UCPVax) associated with atezolizumab for the treatment of locally advanced or metastatic HPV‐positive cancers (HPV16+). Limitations of this study include its retrospective nature, its small size, and the absence of some data on factors that influenced the choice of treatment strategy. The results of this pilot observation should be treated with caution.

## 5 CONCLUSIONS

The optimal management of early‐stage cervical cancer is not currently standardized. Our study, comparing surgery alone versus preoperative radiation therapy, did not identify any difference in DFS (*p* = .858) or OS (*p* = .961) according to the treatment strategy. In the subgroup of patients with ≥IB1 disease, we also failed to show any significant difference in DFS (*p* = .2) or OS (*p* = .2). Cervical cancer counts among the only solid tumors for which increasing mortality has been observed in recent years.^36^ Therefore, key issues remain outstanding for the future management of young patients with cervical cancer and include reducing the morbidity of existing therapeutic options by identifying subgroups of patients at low or intermediate risk and facilitating fertility‐sparing surgery.

## AUTHOR CONTRIBUTIONS

**Katia Mahiou:** Conceptualization (equal); data curation (equal); formal analysis (equal); investigation (equal); methodology (equal); writing – original draft (lead); writing – review and editing (lead). **Laura Vincent:** Supervision (equal); validation (equal). **Karine Peignaux‐Casasnovas:** Supervision (equal); validation (equal). **Hélène Costaz:** Resources (equal). **Marie‐Martine Padeano:** Resources (equal). **Alix Amet:** Resources (equal). **Sandrine Dabakuyo:** Methodology (equal); software (equal); supervision (equal). **Leïla Bengrine‐Lefevre:** Resources (equal). **Charles Coutant:** Supervision (equal); validation (equal). **Ariane MAMGUEM KAMGA:** Software (equal).

## CONFLICT OF INTEREST STATEMENT

This article has not been published in any other newspaper. We do not declare any conflict of interest.

## ETHICS STATEMENT

Ethical and institutional review board approval has been obtained at Georges François Leclerc Cancer Center—UNICANCER, Dijon, France.

## Data Availability

Data sharing is not applicable to this article as no new data were created or analyzed in this study.
